# 1-(2-Hy­droxy­eth­yl)pyrrole-2,5-dione

**DOI:** 10.1107/S1600536812008938

**Published:** 2012-03-07

**Authors:** Xue-Jie Tan, Ting-Wen Du, Dian-Xiang Xing, Yun Liu

**Affiliations:** a School of Chemical and Pharmaceutical Engineering, Shandong Institute of Light Industry, Jinan 250353, People’s Republic of China

## Abstract

The asymmetric unit of the title compound, C_6_H_7_NO_3_, contains two mol­ecules (*A* and *B*) related by a non-crystallographic twofold pseudo-axis. The mol­ecules are joined in the (*AABB*)_*n*_ manner by O—H⋯O hydrogen bonds between their hy­droxy groups, thus forming *C*(2) chains along the *a*-axis direction. Neighboring mol­ecules of the same kind (*A* and *A*, or *B* and *B*) are related by inversion centers, so that all hy­droxy H atoms are disordered other two sets of sites with half occupancies (superimposed O—H⋯O and O⋯H—O fragments). The mol­ecules are further linked by C—H⋯O inter­actions, which can be considered to be weak hydrogen bonds.

## Related literature

For self-initiated photopolymerization, see: Cheng *et al.* (2006 ▶); Ericsson (2001 ▶). For photopolymerization of *N*-substituted maleimides, see: Yamada *et al.* (1968 ▶). For applications of similar compounds, see: Stang & White (2011 ▶); Sanchez *et al.* (2011 ▶); Keller *et al.* (2005 ▶). For the synthesis of the title compound, see: Yamada *et al.* (1961 ▶); Gramlich *et al.* (2010 ▶); Heath *et al.* (2008 ▶).
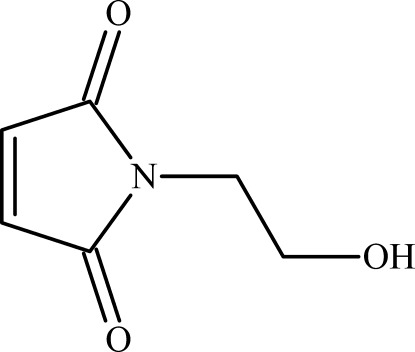


## Experimental

### 

#### Crystal data

C_6_H_7_NO_3_
*M* *_r_* = 141.13
Monoclinic, 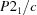

*a* = 7.734 (4) Å
*b* = 9.701 (5) Å
*c* = 17.673 (8) Å
β = 96.660 (7)°
*V* = 1317.0 (11) Å^3^
*Z* = 8
Mo *K*α radiation
μ = 0.12 mm^−1^
*T* = 293 K
0.45 × 0.29 × 0.26 mm

#### Data collection

Bruker SMART CCD diffractometer
Absorption correction: multi-scan (*SADABS*; Bruker, 2000 ▶) *T*_min_ = 0.962, *T*_max_ = 0.976
7522 measured reflections
3003 independent reflections
1972 reflections with *I* > 2σ(*I*)
*R*_int_ = 0.060

#### Refinement

*R*[*F* ^2^ > 2σ(*F* ^2^)] = 0.081
*wR*(*F* ^2^) = 0.217
*S* = 1.10
3003 reflections
201 parameters
8 restraints
H atoms treated by a mixture of independent and constrained refinement
Δρ_max_ = 0.38 e Å^−3^
Δρ_min_ = −0.29 e Å^−3^

### 

Data collection: *SMART* (Bruker, 2000 ▶); cell refinement: *SAINT* (Bruker, 2000 ▶); data reduction: *SAINT*; program(s) used to solve structure: *SHELXTL* (Sheldrick, 2008 ▶); program(s) used to refine structure: *SHELXL97* (Sheldrick, 2008 ▶); molecular graphics: *SHELXTL*; software used to prepare material for publication: *SHELXL97*.

## Supplementary Material

Crystal structure: contains datablock(s) I, global. DOI: 10.1107/S1600536812008938/yk2042sup1.cif

Structure factors: contains datablock(s) I. DOI: 10.1107/S1600536812008938/yk2042Isup2.hkl

Supplementary material file. DOI: 10.1107/S1600536812008938/yk2042Isup3.cml

Additional supplementary materials:  crystallographic information; 3D view; checkCIF report

## Figures and Tables

**Table 1:** Hydrogen-bond geometry (Å, °)

*D*—H⋯*A*	*D*—H	H⋯*A*	*D*⋯*A*	*D*—H⋯*A*
C3*A*—H3*A*⋯O2*B*^iii^	0.93	2.38	3.188 (4)	146
C4*B*—H4*B*⋯O5*A*^i^	0.93	2.49	3.114 (4)	125
O12*A*—H12*A*⋯O12*B*	0.82 (1)	1.91 (1)	2.688 (3)	158 (3)
O12*A*—H12*C*⋯O12*A*^i^	0.82 (1)	2.01 (4)	2.702 (5)	142 (7)
O12*B*—H12*B*⋯O12*A*	0.82 (1)	1.88 (2)	2.688 (3)	168 (8)
O12*B*—H12*D*⋯O12*B*^ii^	0.82 (1)	1.98 (2)	2.773 (4)	163 (5)

Symmetry codes: (i) 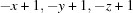; (ii) 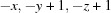; (iii) 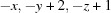.

**Fig. 1.**
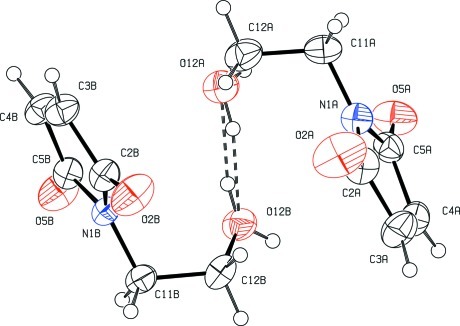
The asymmetric unit of the title compound with atom labelling scheme and thermal ellipsoids drawn at the 40% probability level. Intermolecular hydrogen bonds O—H···O are presented by dashed lines.

**Fig. 2.**
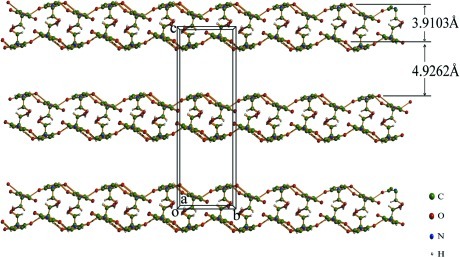
Portion of six infinite two-dimensional corrugated sheets in (I) linked by hydrogen-bonds, viewed along the a axis. These six sheets can be dubbed in three pairs of hydrogen-bonded layers.

## supplementary crystallographic information

### Comment

Maleimides are a class of reactive "synthons" having a polymerizable double bond. They are particularly useful in manufacturing oligomers capable of self-initiated photopolymerization (Cheng *et al.*, 2006; Ericsson, 2001). The title compound, *N*-2-hydroxyethylmaleimide, first prepared in 1961 (Yamada *et al.*, 1961), is a well-known maleimide that has been intensively studied during last years (Stang & White, 2011; Sanchez *et al.*, 2011; Keller *et al.*, 2005). However its crystal structure has not been determined. In this work, the crystal structure of the title compound is reported, and its molecular packing mode is discussed.

As shown in Fig. 1, the asymmetric unit of the title compound contains two molecules (A and B) related by the non-crystallographic two-fold pseudo-axis. The molecules are joined in the (AABB)_n_ manner by O—H···O hydrogen bonds between their hydroxy groups, thus forming the C(2) chains stretched along the *a*-axis direction. The neighboring molecules of the same kind (A and A, or B and B) are related by inversion centers, so that all hydroxy hydrogen atoms are disordered other two sets of sites with half occupancies, thus the fragments O—H···O and O···H—O are superimposed. The molecules are further linked by intermolecular C—H···O interactions, which can be considered as weak hydrogen bonds.

Instead of helices, hydrogen bonds make (I) pack into zigzag-type pleated sheets stretched along (0 0 1) planes (Fig. 2). Adjacent sheets are arranged in an antiparallel manner, yielding an ABAB layer sequence. Either O—H···O and C—H···O interactions or no such interactions occur between adjacent sheets. As can be seen, the hydrogen-bonded sheets are rather closely spaced in the lattice (3.9103 (9) Å) than no-hydrogen-bonded sheets (4.9262 (8) Å).

### Experimental

The title compound was synthesized using established method (Gramlich *et al.*, 2010; Heath *et al.*, 2008). Elemental analysis: Calcd: C 51.06; H 5.00; N 9.93%. Found: C 51.11; H 4.92; N 10.02%.

### Refinement

The C-bound H atoms were placed in calculated positions with C—H = 0.93–0.97 Å and allowed to ride on their parent atoms with *U*_iso_(H) = 1.2*U*_eq_(C). The disordered O-bound H atoms with half occupancies were refined with the O—H and C···H distances restrained to 0.82 (1) Å and 1.85 (2) Å and with *U*_iso_(H) = 1.5*U*_eq_(O).

### Crystal data

**Table:**

C_6_H_7_NO_3_	*F*(000) = 592.0
*M**_r_* = 141.13	*D*_x_ = 1.424 Mg m^−^^3^
Monoclinic, *P*2_1_/*c*	Melting point: 344 K
Hall symbol: -P 2ybc	Mo *K*α radiation, λ = 0.71073 Å
*a* = 7.734 (4) Å	Cell parameters from 380 reflections
*b* = 9.701 (5) Å	θ = 2.5–28.3°
*c* = 17.673 (8) Å	µ = 0.12 mm^−^^1^
β = 96.660 (7)°	*T* = 293 K
*V* = 1317.0 (11) Å^3^	Block, colourless
*Z* = 8	0.45 × 0.29 × 0.26 mm

### Data collection

**Table:**

Bruker SMART CCD diffractometer	3003 independent reflections
Radiation source: fine-focus sealed tube	1972 reflections with *I* > 2σ(*I*)
Graphite monochromator	*R*_int_ = 0.060
φ and ω scans	θ_max_ = 28.4°, θ_min_ = 2.3°
Absorption correction: multi-scan (*SADABS*; Bruker, 2000)	*h* = −8→10
*T*_min_ = 0.962, *T*_max_ = 0.976	*k* = −11→12
7522 measured reflections	*l* = −23→23

### Refinement

**Table:**

Refinement on *F*^2^	Primary atom site location: structure-invariant direct methods
Least-squares matrix: full	Secondary atom site location: difference Fourier map
*R*[*F*^2^ > 2σ(*F*^2^)] = 0.081	Hydrogen site location: inferred from neighbouring sites
*wR*(*F*^2^) = 0.217	H atoms treated by a mixture of independent and constrained refinement
*S* = 1.10	*w* = 1/[σ^2^(*F*_o_^2^) + (0.097*P*)^2^ + 0.420*P*] where *P* = (*F*_o_^2^ + 2*F*_c_^2^)/3
3003 reflections	(Δ/σ)_max_ = 0.001
201 parameters	Δρ_max_ = 0.38 e Å^−^^3^
8 restraints	Δρ_min_ = −0.29 e Å^−^^3^

### Special details

**Table:**

Geometry. All s.u.'s (except the s.u. in the dihedral angle between two l.s. planes) are estimated using the full covariance matrix. The cell s.u.'s are taken into account individually in the estimation of s.u.'s in distances, angles and torsion angles; correlations between s.u.'s in cell parameters are only used when they are defined by crystal symmetry. An approximate (isotropic) treatment of cell s.u.'s is used for estimating s.u.'s involving l.s. planes.
Refinement. Refinement of *F*^2^ against ALL reflections. The weighted *R*-factor *wR* and goodness of fit *S* are based on *F*^2^, conventional *R*-factors *R* are based on *F*, with *F* set to zero for negative *F*^2^. The threshold expression of *F*^2^ > σ(*F*^2^) is used only for calculating *R*-factors(gt) *etc*. and is not relevant to the choice of reflections for refinement. *R*-factors based on *F*^2^ are statistically about twice as large as those based on *F*, and *R*- factors based on ALL data will be even larger.

### Fractional atomic coordinates and isotropic or equivalent isotropic displacement parameters (Å^2^)

**Table:**

	*x*	*y*	*z*	*U*_iso_*/*U*_eq_	Occ. (<1)
O2A	0.2520 (4)	1.0076 (3)	0.5604 (2)	0.0911 (10)	
O5A	0.2601 (3)	0.5693 (2)	0.64576 (16)	0.0653 (7)	
O12A	0.4289 (3)	0.6222 (3)	0.47672 (15)	0.0617 (7)	
H12A	0.3228 (14)	0.630 (4)	0.475 (5)	0.093*	0.50
H12B	0.1884 (13)	0.594 (5)	0.452 (5)	0.093*	0.50
O2B	0.2505 (3)	0.9305 (2)	0.33843 (17)	0.0709 (8)	
O5B	0.2400 (3)	0.4751 (2)	0.28146 (15)	0.0622 (7)	
O12B	0.0831 (3)	0.5827 (2)	0.45141 (14)	0.0529 (6)	
H12C	0.450 (9)	0.561 (3)	0.508 (2)	0.079*	0.50
H12D	0.023 (5)	0.548 (6)	0.481 (4)	0.079*	0.50
N1A	0.3035 (3)	0.7840 (2)	0.59889 (14)	0.0440 (6)	
N1B	0.1968 (3)	0.7013 (2)	0.31464 (13)	0.0369 (6)	
C2A	0.2026 (5)	0.9003 (3)	0.5835 (2)	0.0544 (8)	
C3A	0.0255 (5)	0.8636 (4)	0.6022 (2)	0.0607 (9)	
H3A	−0.0708	0.9216	0.5973	0.073*	
C4A	0.0275 (4)	0.7364 (4)	0.62661 (18)	0.0527 (8)	
H4A	−0.0669	0.6888	0.6420	0.063*	
C5A	0.2058 (4)	0.6808 (3)	0.62565 (17)	0.0435 (7)	
C11A	0.4858 (4)	0.7719 (3)	0.5876 (2)	0.0510 (8)	
H111	0.5380	0.6972	0.6189	0.061*	
H112	0.5458	0.8564	0.6040	0.061*	
C12A	0.5090 (4)	0.7447 (4)	0.5057 (2)	0.0583 (9)	
H121	0.4612	0.8216	0.4751	0.070*	
H122	0.6325	0.7398	0.5009	0.070*	
C2B	0.2981 (4)	0.8183 (3)	0.32075 (18)	0.0436 (7)	
C3B	0.4715 (4)	0.7761 (3)	0.30062 (19)	0.0483 (8)	
H3B	0.5671	0.8340	0.2997	0.058*	
C4B	0.4683 (4)	0.6462 (3)	0.28466 (18)	0.0468 (8)	
H4B	0.5613	0.5957	0.2702	0.056*	
C5B	0.2936 (4)	0.5904 (3)	0.29288 (17)	0.0412 (7)	
C11B	0.0143 (4)	0.6925 (3)	0.32856 (17)	0.0432 (7)	
H113	−0.0359	0.6087	0.3055	0.052*	
H114	−0.0489	0.7700	0.3041	0.052*	
C12B	−0.0084 (4)	0.6927 (3)	0.41195 (19)	0.0491 (8)	
H123	0.0332	0.7795	0.4343	0.059*	
H124	−0.1313	0.6850	0.4177	0.059*	

### Atomic displacement parameters (Å^2^)

**Table:**

	*U*^11^	*U*^22^	*U*^33^	*U*^12^	*U*^13^	*U*^23^
O2A	0.089 (2)	0.0435 (16)	0.144 (3)	0.0012 (14)	0.0289 (19)	0.0214 (17)
O5A	0.0582 (15)	0.0482 (14)	0.0865 (19)	−0.0029 (11)	−0.0048 (13)	0.0229 (13)
O12A	0.0487 (13)	0.0734 (17)	0.0628 (16)	0.0004 (12)	0.0055 (12)	−0.0066 (12)
O2B	0.0717 (17)	0.0406 (14)	0.105 (2)	0.0016 (11)	0.0321 (15)	−0.0125 (13)
O5B	0.0615 (15)	0.0379 (13)	0.0905 (19)	−0.0007 (10)	0.0232 (13)	−0.0092 (12)
O12B	0.0448 (12)	0.0588 (14)	0.0561 (14)	−0.0039 (11)	0.0105 (11)	0.0157 (11)
N1A	0.0459 (14)	0.0391 (14)	0.0468 (14)	−0.0010 (11)	0.0048 (11)	0.0041 (11)
N1B	0.0362 (12)	0.0353 (13)	0.0407 (13)	0.0040 (10)	0.0109 (10)	0.0037 (10)
C2A	0.066 (2)	0.0370 (18)	0.060 (2)	0.0050 (15)	0.0101 (17)	0.0021 (15)
C3A	0.060 (2)	0.053 (2)	0.072 (2)	0.0157 (16)	0.0204 (18)	−0.0029 (18)
C4A	0.0535 (19)	0.061 (2)	0.0456 (18)	−0.0009 (16)	0.0136 (15)	−0.0041 (15)
C5A	0.0464 (17)	0.0445 (17)	0.0388 (15)	−0.0010 (13)	0.0011 (12)	0.0060 (13)
C11A	0.0379 (16)	0.0485 (18)	0.065 (2)	−0.0061 (14)	0.0005 (14)	−0.0005 (16)
C12A	0.0439 (18)	0.062 (2)	0.071 (2)	−0.0044 (16)	0.0140 (16)	0.0094 (18)
C2B	0.0481 (17)	0.0371 (16)	0.0468 (17)	0.0009 (13)	0.0109 (13)	0.0052 (13)
C3B	0.0433 (17)	0.0446 (18)	0.0588 (19)	−0.0076 (13)	0.0133 (14)	0.0083 (15)
C4B	0.0428 (17)	0.0435 (17)	0.0567 (19)	0.0110 (13)	0.0170 (14)	0.0131 (14)
C5B	0.0479 (17)	0.0338 (16)	0.0427 (16)	0.0073 (12)	0.0089 (13)	0.0064 (12)
C11B	0.0368 (15)	0.0454 (17)	0.0484 (17)	0.0037 (12)	0.0092 (13)	0.0045 (13)
C12B	0.0490 (18)	0.0458 (18)	0.0565 (19)	0.0015 (14)	0.0227 (15)	0.0005 (15)

### Geometric parameters (Å, º)

**Table:**

O2A—C2A	1.196 (4)	C3A—H3A	0.9300
O5A—C5A	1.199 (4)	C4A—C5A	1.483 (4)
O12A—C12A	1.409 (4)	C4A—H4A	0.9300
O12A—H12A	0.821 (10)	C11A—C12A	1.503 (5)
O12A—H12C	0.821 (10)	C11A—H111	0.9700
O2B—C2B	1.202 (4)	C11A—H112	0.9700
O5B—C5B	1.201 (4)	C12A—H121	0.9700
O12B—C12B	1.418 (4)	C12A—H122	0.9700
O12B—H12B	0.821 (10)	C2B—C3B	1.484 (4)
O12B—H12D	0.817 (10)	C3B—C4B	1.291 (4)
N1A—C5A	1.371 (4)	C3B—H3B	0.9300
N1A—C2A	1.381 (4)	C4B—C5B	1.478 (4)
N1A—C11A	1.452 (4)	C4B—H4B	0.9300
N1B—C2B	1.376 (4)	C11B—C12B	1.504 (4)
N1B—C5B	1.391 (4)	C11B—H113	0.9700
N1B—C11B	1.463 (4)	C11B—H114	0.9700
C2A—C3A	1.489 (5)	C12B—H123	0.9700
C3A—C4A	1.307 (5)	C12B—H124	0.9700
			
C12A—O12A—H12A	110 (2)	O12A—C12A—C11A	113.7 (3)
C12A—O12A—H12C	109 (2)	O12A—C12A—H121	108.8
H12A—O12A—H12C	102 (8)	C11A—C12A—H121	108.8
C12B—O12B—H12B	110 (2)	O12A—C12A—H122	108.8
C12B—O12B—H12D	110 (2)	C11A—C12A—H122	108.8
H12B—O12B—H12D	133 (6)	H121—C12A—H122	107.7
C5A—N1A—C2A	110.1 (3)	O2B—C2B—N1B	125.3 (3)
C5A—N1A—C11A	124.8 (3)	O2B—C2B—C3B	128.6 (3)
C2A—N1A—C11A	125.1 (3)	N1B—C2B—C3B	106.0 (2)
C2B—N1B—C5B	109.9 (2)	C4B—C3B—C2B	109.1 (3)
C2B—N1B—C11B	125.9 (2)	C4B—C3B—H3B	125.5
C5B—N1B—C11B	124.2 (2)	C2B—C3B—H3B	125.5
O2A—C2A—N1A	125.6 (3)	C3B—C4B—C5B	109.3 (3)
O2A—C2A—C3A	128.5 (3)	C3B—C4B—H4B	125.3
N1A—C2A—C3A	105.9 (3)	C5B—C4B—H4B	125.3
C4A—C3A—C2A	108.9 (3)	O5B—C5B—N1B	125.5 (3)
C4A—C3A—H3A	125.6	O5B—C5B—C4B	128.7 (3)
C2A—C3A—H3A	125.6	N1B—C5B—C4B	105.7 (2)
C3A—C4A—C5A	108.4 (3)	N1B—C11B—C12B	112.9 (3)
C3A—C4A—H4A	125.8	N1B—C11B—H113	109.0
C5A—C4A—H4A	125.8	C12B—C11B—H113	109.0
O5A—C5A—N1A	125.0 (3)	N1B—C11B—H114	109.0
O5A—C5A—C4A	128.2 (3)	C12B—C11B—H114	109.0
N1A—C5A—C4A	106.8 (3)	H113—C11B—H114	107.8
N1A—C11A—C12A	111.9 (3)	O12B—C12B—C11B	111.9 (2)
N1A—C11A—H111	109.2	O12B—C12B—H123	109.2
C12A—C11A—H111	109.2	C11B—C12B—H123	109.2
N1A—C11A—H112	109.2	O12B—C12B—H124	109.2
C12A—C11A—H112	109.2	C11B—C12B—H124	109.2
H111—C11A—H112	107.9	H123—C12B—H124	107.9

### Hydrogen-bond geometry (Å, º)

**Table:**

*D*—H···*A*	*D*—H	H···*A*	*D*···*A*	*D*—H···*A*
O12*A*—H12*A*···O12*B*	0.82 (1)	1.91 (1)	2.688 (3)	158 (3)
O12*B*—H12*B*···O12*A*	0.82 (1)	1.88 (2)	2.688 (3)	168 (8)
O12*A*—H12*C*···O12*A*^i^	0.82 (1)	2.01 (4)	2.702 (5)	142 (7)
O12*B*—H12*D*···O12*B*^ii^	0.82 (1)	1.98 (2)	2.773 (4)	163 (5)
C4*B*—H4*B*···O5*A*^i^	0.93	2.49	3.114 (4)	125
C3*A*—H3*A*···O2*B*^iii^	0.93	2.38	3.188 (4)	146

Symmetry codes: (i) −*x*+1, −*y*+1, −*z*+1; (ii) −*x*, −*y*+1, −*z*+1; (iii) −*x*, −*y*+2, −*z*+1.
